# Influence of Process Parameters in Material Extrusion on Product Properties Using the Example of the Electrical Resistivity of Conductive Polymer Composites

**DOI:** 10.3390/polym15224452

**Published:** 2023-11-17

**Authors:** Maximilian Nowka, Karl Hilbig, Lukas Schulze, Eggert Jung, Thomas Vietor

**Affiliations:** Institute for Engineering Design, Technische Universität Braunschweig, Hermann-Blenk-Str. 42, 38108 Brunswick, Germany; k.hilbig@tu-braunschweig.de (K.H.); lukas.schulze@tu-braunschweig.de (L.S.); t.vietor@tu-braunschweig.de (T.V.)

**Keywords:** additive manufacturing, material extrusion, 3D printing, electrically conductive, conductive polymer composite, filament fabrication, design for additive manufacturing, electrical resistivity, Fused Deposition Modeling

## Abstract

Additive manufacturing of components using the material extrusion (MEX) of thermoplastics enables the integration of multiple materials into a single part. This can include functional structures, such as electrically conductive ones. The resulting functional structure properties depend on the process parameters along the entire manufacturing chain. The aim of this investigation is to determine the influence of process parameters in filament production and additive manufacturing on resistivity. Filament is produced from a commercially available composite of polylactide (PLA) with carbon nanotubes (CNT) and carbon black (CB), while the temperature profile and screw speed were varied. MEX specimens were produced using a full-factorial variation in extrusion temperature, layer height and deposition speed from the most and least conductive in-house-produced filament and the commercially available filament from the same composite. The results show that the temperature profile during filament production influences the resistivity. The commercially available filament has a lower conductivity than the in-house-produced filament, even though the starting feedstock is the same. The process parameters during filament production are the main factors influencing the resistivity of an additively manufactured structure. The MEX process parameters have a minimal influence on the resistivity of the used PLA/CNT/CB composite.

## 1. Introduction

Additive manufacturing (AM), often referred to as 3D printing, opens up new possibilities in product design. The layer-by-layer manufacturing process has the potential to offer an advantage over subtractive manufacturing processes in niche applications, despite the higher costs. On the one hand, the freedom of shape is almost unrestricted since the complexity of the shape does not significantly influence the process costs. This means, for example, that undercuts or geometrically determined grid structures can be used in the design process. On the other hand, additive manufacturing processes offer a high degree of material freedom, which, depending on the process, can be used in combination as a multi-material part design. This design approach enables the integration of several functions, such as electrically conductive or flexible structures, through the use of functional polymers. The material properties are influenced by the layered structure, resulting in strongly anisotropic properties. These are a challenge, especially for the mechanical and electrical component properties, but can also be utilized as a design flexibility through sufficient knowledge [1].

One of the most widespread additive manufacturing processes is thermal material extrusion (MEX) [2]. This process uses thermoplastic feedstock in the form of powder, granulate and plastic wires (filament) [3]. The properties of the matrix polymer can be significantly influenced by adding additives and fillers [4]. For example, the addition of carbon allotropes in the form of carbon black, graphene, or carbon nanostructures can change the electrical properties of the polymer from insulating to conductive [5].

**Figure 1:**
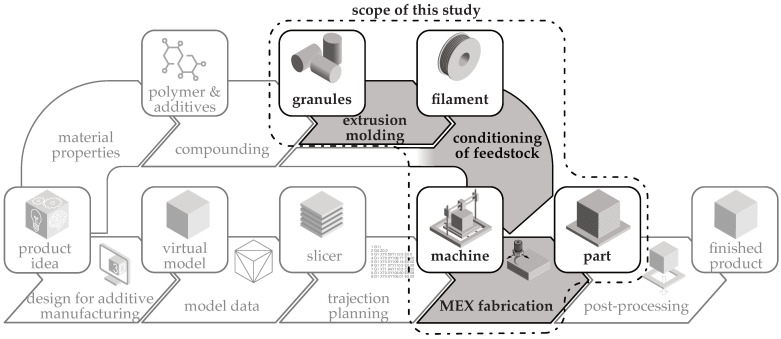
Scope of this study in the general additive manufacturing process chain, considering the material processing of the feedstock.

The figure illustrates the AM process chain, which is extended to include material processing. The specific scope of this publication is highlighted in Figure 1.

This study uses the commercially available material ALFAOHM (FILOALFA^®^ by Ciceri de Model Srl (further shortened to FILOALFA^®^), Ozzero, Itlay) [6] to compare the manufacturing influences on the electrical properties of the filament and the resulting electrical component properties of MEX structures.

## 2. State of the Art

This section reviews the state of the art in material extrusion and the resulting anisotropic part properties. An overview of the electrically conductive feedstock products available for the MEX process is presented. There will also be a discussion of the factors that have been the focus of research into the influences on conductivity.

### 2.1. Material Extrusion in Multi-Material Part Design

Additive manufacturing processes produce parts with a defined shape from shapeless materials, in particular liquids, granules, powders, or wires, through the use of energy or binding agents and are assigned to the primary shaping processes according to DIN 8580 [7]. The material extrusion process used here (formerly Fused Deposition Modeling (FDM), Fused Filament Fabrication (FFF), or Fused Layer Manufacturing (FLM)) is referred to as MEX-TRB/P in accordance with DIN EN ISO 52900 and is abbreviated to MEX in the following [3]. The feedstock is a thermoplastic polymer (P) deposited by material extrusion (MEX). The layered adhesion is formed by thermal reaction bonding (TRB) [3,7].

In the MEX process, plasticized polymer melt is extruded from the heated nozzle [2,4,8,9]. The nozzle is located at a distance of one layer height from the part [2,9]. The deposited strands weld directly with the neighboring strands or the strands underneath. This connection is formed by melting the surface of the already solidified strands due to the hot newly deposited strands [2,10]. The relative position of the nozzle to the building platform, and also to the part to be manufactured, is controlled by the machine control [2,8]. During the manufacturing process, a layer is created by depositing strands. The strand-by-strand construction of a layer, as well as the layer-by-layer construction of parts, leads to strongly anisotropic part properties. The anisotropy is particularly evident in the mechanical strength characteristics and, in the case of electrically conductive thermoplastics, in the electrical conductivity [2,4,8].

**Figure 2:**
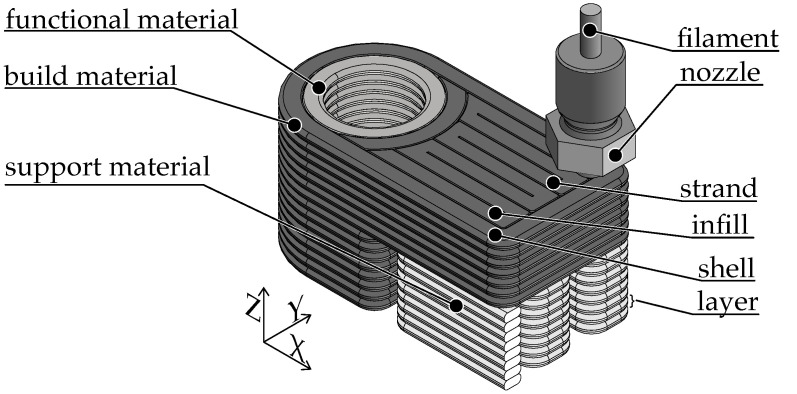
Manufacturing process of a part using the MEX manufacturing process in the multi-material design.

The selective application of materials within a layer makes it possible to combine different materials [11]. The used thermoplastics can be classified into one of three groups with regard to their application area within the multi-material part. The product geometry is determined by the main structure, which is made of the build material [2,4,10,11]. Structures made of functional polymers are integrated into the main structure at relevant locations, which enable the additional product function due to the specific material properties [10,12,13]. For the manufacturing process, it may be necessary to produce temporary support structures, e.g., for overhangs from the build material. The support material can be removed mechanically or chemically in a post-processing step [2,4,8,10,11]. Figure 2 shows an example part in a multi-material design consisting of a build material, a functional material for the cylindrical functional surface and a support material for the L-shaped overhang.

#### 2.1.1. Material Extrusion—Filament

ISO 52900 uses the term MEX to refer to a number of process variants, such as extrusion of granulate and filament [3]. The filament extrusion process is based on the processing of a wire-like plastic feedstock, the so-called filament. This is conveyed by a feed mechanism into a hotend heated above the melting point of the thermoplastic. The plastic is plasticized in the hotend and extruded from the nozzle as a thin viscous strand by the continuous feeding of filament. The process reliability of the MEX process depends, in particular, on the geometric quality of the filament. The filament feed in the filament extruder is based on the assumption of a constant diameter and round geometry, so any deviation will result in the extrusion of too much (over-extrusion) or too little (under-extrusion) material [4,10].

#### 2.1.2. Material Extrusion—Fabrication of Filament as a Feedstock

The filament-based MEX variant requires a geometrically accurate plastic wire as a feedstock. This is produced by a continuous extrusion process. A plastic extruder plasticizes the granules due to the heat and mechanical friction generated by the screw. At the end of the screw, the polymer melt is forced through the nozzle in a highly viscous state. By adjusting the pull-off speed at which the polymer melt is pulled from the die, the diameter can be thinned to a certain degree by the drawing process. The hot viscous melt is cooled to ambient temperature in water. The temperature of the water influences the solidification behavior of the polymer melt. Due to the similar densities of water and plastics, the solidification process takes place with a reduced effect of weight, maintaining the round shape of the filament. After solidification, an inline inspection of the geometry is performed before the pull-off unit. In the final process step, the material is passed through a material buffer and spooled onto standardized rolls [4].

Due to the wide range of machines and their combinations in filament production plants, e.g., the extruders and water basins, it is not possible to directly transfer the results with the same plant process parameters to other plants.

### 2.2. Resistivity in the Context of Material Extrusion

The electrical resistance R is a physical dimension that is the measure of the restriction of the current flow I at an applied voltage U. This relationship is described by Ohm’s law:

(1) U=R·I

The resistance R of a conducting structure depends on the resistivity 𝜌 and its geometric dimensions, the length L, and the cross-section:

(2) $$R=\rho \cdot \frac{L}{A}=\frac{1}{\sigma }\cdot \frac{L}{A}$$

The reciprocal resistivity is the conductivity σ. The processing of electrically isotropic materials with conventional processing methods, for example, machining, also leads to electrically isotropic part properties. When using the MEX process, the strand- and layer-wise production results in direction-dependent electrical properties.

### 2.3. The Research Gap

Numerous studies have demonstrated that parameters in MEX processing, such as temperature and shear rate, can influence the resistivity of additively manufactured structures [14,15]. However, previous publications in the field of manufacturing electrically conductive filaments have primarily focused on the production of feedstock from in-house-produced materials for further processing [16,17]. The factors influencing electrical conductivity during filament production have rarely been investigated [14,18,19]. This can be seen in the overview Table 1.

Gonçalves et al. influenced the electrical conductivity in preliminary tests by varying the screw geometry, but did not describe it further [19].

In order to produce a dimensionally accurate filament, Podsiadły et al. adjusted the temperature profile of a single-screw extruder to the flow behavior of the in-house-produced polymer composite. No analysis of the influence of the temperature profile during filament production on conductivity was carried out [18].

The most comprehensive study was carried out by Paz et al. In this study, the temperatures, die geometry, and screw speed were each investigated with two variations. It was demonstrated that the process parameters during filament production influence the conductivity of the filament. Nevertheless, no conclusion can be drawn about the effects of the influencing factors between the data points, as only two are examined in each variation. Furthermore, only the most conductive material was used for the additive manufacturing of the specimens. Therefore, it is impossible to predict the influence of the process parameters during filament production on the electrical conductivity of an additively manufactured structure [14].

To date, a systematic analysis of the influences of process parameters during filament production on the electrical conductivity of additively manufactured electrical functional structures has not been carried out. The aim of this study is to produce filaments with more variations in the influencing factors than Paz et al. and to produce MEX specimens with the most and least conductive in-house filament as well as an industrial produced filament as reference [14]. Fabricating a specimen using both most and least conductive filaments provides an indication of whether the different conductivity of the feedstock has the same effect on the conductivity of the MEX structures. The production of additional specimen from the commercially available filament enables possible comparisons with future studies.

## 3. Materials and Methods

In the following section, the equipment for the production of filament and MEX specimens, the used feedstock, the approach as well as the Design of Experiment are presented.

### 3.1. Overview of the Production and Measurement Steps

**Figure 3:**
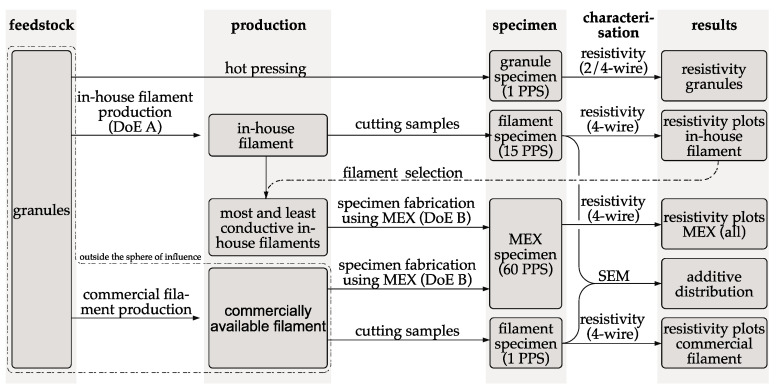
Schematic overview of the phases in this study of the fabrication of feedstock, as well as specimen and characterization (PPS = process parameter set).

This study investigates the effects of the process parameters on the electrical conductivity during the filament production and additively manufactured structures. Figure 3 visualizes the procedure of the manufacturing steps and characterizations of the process parameters during filament production on the electrical conductivity of filament and additively manufactured structures are investigated.

ALFAOHM granules are sourced from the company FILOALFA^®^ (Ozzero, Italy). FILOALFA^®^ itself uses these granules for the production of their commercially distributed ALFAOHM filament. The granules are the starting point of the process chain and are therefore also examined with regard to resistivity. Filament is produced from the granules according to DoE A with different process parameter sets (PPS). For each resulting process parameter set, the filament is characterized with regard to its resistivity. As a benchmark, the filament commercially produced by FILOALFA^®^ is also characterized in the same way. For the MEX processing step, the most, and least conductive in-house filament are used, as well as filament produced by FILOALFA^®^. The input factors for the MEX specimens are listed in DoE B. The specimens are then characterized with a 4-wire measurement and the resistivity is calculated from the geometric dimensions and the measured resistance according to Equation (2). SEM images are taken of selected filament samples and MEX specimens to evaluate the filler distribution.

### 3.2. Overview of the Manufacturing Processes Used

For the production within the two specific manufacturing routes, different production plants were used. A self-developed production line has been used for the extrusion molding process in filament production. The developed production line is based on the production steps of industrial filament production lines. The process has been scaled down to a laboratory scale, enabling the processing of small quantities of granules (<1 kg) into a usable quantity of filament.

**Figure 4:**
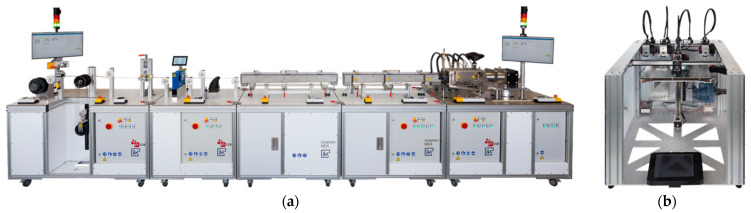
Equipment used to produce MEX structures from granules: (**a**) filament production plant for the extrusion molding process of filament; (**b**) additive manufacturing machine (Toolchanger- e3d^®^) for the production of specimen using MEX.

The filament production plant (see Figure 4a) offers the possibility of producing filaments from small amounts of bulk material on a laboratory scale. The extruder has five zones, of which four are heated. The screw has a diameter of 20 mm and an L/D ratio of 14.5. The extruder is equipped with a circular die with a diameter of 2 mm.

The roundness of the filament is measured inline during the manufacturing process using a laser diameter-measuring instrument (Zumbach ODAC 13 TRIO, (Zumbach Electronic AG, Orpund, Switzerland)) with a sampling rate of 3 Hz, in three axes, with a resolution of 0.1 μm. The length of both water basins is 1000 mm. The temperature of the hot water basin is adjusted according to the polymer composite used. The temperature of the cold-water basin is constant at room temperature at 23 °C during the production process. The spooling process of the filament starts after the process has reached the quality requirements for geometric consistency.

For the production of specimens from filament through the MEX process, a machine (ToolChanger e3d^®^, (E3D-Online, Chalgrove, Oxfordshire, UK)) with direct drive extruders (Hemera e3d^®^, (E3D-Online, Chalgrove, Oxfordshire, UK)) for 1.75 mm filament and hardened steel nozzles (Nozzle X e3d^®^, (E3D-Online, Chalgrove, Oxfordshire, UK)) with a diameter of 0.4 mm is used (see Figure 4b).

### 3.3. Selection of a Commercially Available Electrically Conductive Filament

The widespread use of MEX machines has led to a high diversity of materials. In recent years, various electrically conductive filaments with different properties have been available on the market. Table 2 lists the electrically conductive filaments with a comparison of the conductive additive used, the electrical conductivity, and the operation temperature.

The selection is based on the criteria of availability of the feedstock, both in form of filaments and granules, operating temperature, and conductivity. BlackMagic Conductive, Functionalize F -Electric™ PLA, Blackmagic Conductive flexible filament, and PI-ETPU 85-700+ were not available during the planning of this study. Due to the low heat deflection temperature of the matrix polymer PCL, Multi3d Electrifi is not suitable for technical applications [15]. Due to their low Young’s modulus, both TPU-based filaments, Conductive Filaflex and Eel 3D Printing Filament, are unsuitable for simple contacting and thus for the production of many samples [40]. Amolen conductive PLA has a high conductivity, according to the manufacturer, but the conductive additives are unknown. For this reason, an interpretation of the results with this material is impossible, and it is not suitable for this study. The remaining three most conducting ones include Koltron G1, ALFAOHM and Ampere PLA. Preliminary tests have shown that samples made from ALFAOHM have the lowest resistivity. Therefore, ALFAOHM is used for the experiment. In addition, ALFAOHM is available as granulate and as filament.

The manufacturer FILOALFA^®^ recommends the process parameter limits for ALFAOHM given in Table 3. The limits are used as a baseline for the DoE A and B.

### 3.4. Design of Experiment

For the two-stage processing of filament production and MEX specimen manufacturing, a full-factorial Design of Experiment (DoE) with five specimens per parameter set is designed. The following Table 4 shows the production routes with their input factors.

The input factors of the screw speed and the temperature profile are selected for the filament production DoE A. The temperature profile is linear and is derived from the temperature of the die zone. For example, if the die zone is at 180 °C, the linear temperature profile will be: 180 °C (100%)/135 °C (75%)/90 °C (50%)/45 °C (25%)/22 °C (room temperature).

Previous investigations have focused on the influence of the infill orientation, the extrusion temperature and the layer height on the resistivity (see Table 1). These are used as input factors for DoE B.

The DoE A results in 15 process parameter sets with 75 specimens for filament production and DoE B for MEX in 60 process parameter sets with three filaments (900 specimens).

## 4. Characterization Methods

This section is an introduction to the characterization methods used. There will also be a description of the development of specimens for additive manufacturing on the basis of standardized specimens and the validation of the suitability of the developed specimens. The following methods are used to characterize the feedstock (granules and filament) and the specimens fabricated by MEX:

1. Resistance measurements (2- and 4-wire) of granules, filament and MEX specimens with Keithley 2460 (Keithley Instruments, Solon, OH, USA).
2. Roughness determination of MEX specimens with Keyence VR-5100 profilometer (Keyence, Neu-Isenburg, Germany).
3. Thermal Images with TIM640 with 30° default optic (Micro-Epsilon Messtechnik GmbH & Co. KG, Ortenburg, Germany).
4. SEM and optical microscope images of fracture edges with FEI Helios G4 CX and Keyence VHX-7000 with VH-Z20R optic (FEI, Hillsboro, OR, USA), (Keyence, Neu-Isenburg, Germany).

### 4.1. Diameter of Filament

The filament diameter and the geometrical deviations from an ideal round filament are a particularly important criteria for the process reliability in the MEX process [4,10]. Geometric deviations cause the volume of molten polymer to be either too low or too high [4].

A three-axis diameter sensor ODAC 13TRIO is used for the in situ characterization of the diameter. The resolution is 0.1 µm, the measurement deviation ±0.5 µm and the 3σ repeatability with 0.12 µm. Due to the three-axis scanning of the filament, the ovality can be determined with a small error [41].

Prior to the start of the filament spooling process, some system parameters regarding filament diameter and ovality are carefully tuned. The temperature in the hot water basin is adjusted once for the average value of the process parameters (extrusion temperature: 200 °C, screw speed: 15 rpm). All filaments are produced at a hot water temperature of 80 °C. The pull-off speed is adjusted individually for each screw speed. The spooling process is started when the diameter variations are within ±50 µm around the nominal diameter. Filament sections that do not fulfill these requirements are discarded and not used any further. Maintaining the exact nominal diameter of 1.75 mm is not critical for this study as long as the diameter is within the range of 1.65 mm to 1.75 mm and remains within permissible diameter variations. Deviations from the nominal diameter are compensated for in the following MEX by the process parameter of the flow rate.

### 4.2. Electrical Characterization

There is no dedicated standard for measuring the resistivity of feedstock for MEX or electrically conductive structures additively manufactured using MEX. For this reason, the resistivity measurements are conducted in accordance with ISO 10350-1 in compliance with DIN EN ISO 3915:2022-5, which applies to polymer composites with a resistivity of less than 10^6^ Ω cm [42,43].The standard is only applicable to electrically conductive polymer composites with isotropic resistivity. It was therefore necessary to make the following changes to the procedure for the measurement, which are described in more detail for each experiment:

- The use of planar electrodes is not possible.
- Different geometry of the specimen.

The following aspects are applicable to MEX specimens without change and can therefore be incorporated:

- Specimens with a deviation of more than 5% from the nominal dimension of the adapted specimen are scrapped. By measuring the dimensions with a micrometer screw QuantuMike^®^ 293-140-30 (Mitutoyo Corporation, Kawasaki, Japan) smaller deviations are accounted for when calculating the resistivity.
- The spacing between the contacts is known to be far more precise than 0.2%.
- The contacts on all specimens made of silver paste EMS #12640 (Electron Microscopy Sciences, Hatfield, PA, USA) are used as electrical bonding agents perpendicular to the direction of current flow (both force and sense lines) [44].
- The time between specimen preparation and measurement is more than 16 h.
- The specimens are fabricated on insulating glass slides. Thus, the substrate for the measurement setup has a volume resistance greater than 10^15^ Ω. The specimens are not removed from them and no mechanical stresses are introduced after fabrication.
- The Keithley 2460 sourcemeter is used as a current source so that current can be determined much more accurately than 5% of nominal current.
- The Current is limited to 100 µA and the voltage to 5 V to keep power dissipation below 100 mW.

#### 4.2.1. Measurement of Granules

**Figure 5:**
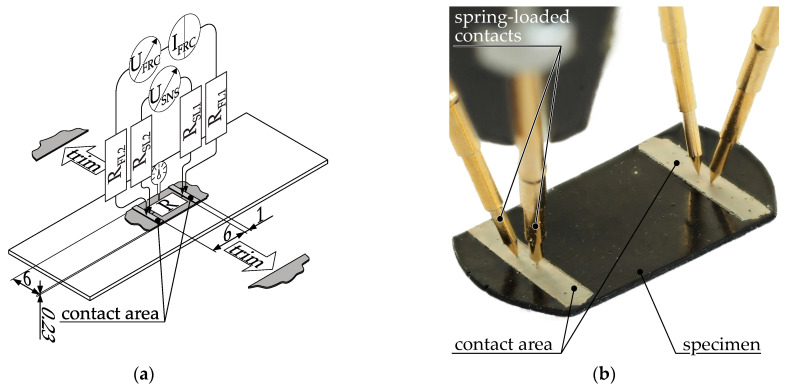
Measurement setup for determining the resistivity of granules: (**a**) schematic illustration and wiring of the experimental setup. R = resistance of granule specimen, R_FL_= resistance force lead, R_SL_ = resistance sense lead; I_FRC_ = forced current, U_FRC_ = voltage of forced current, U_SNS_ = measured voltage drop across sample; (**b**) physical setup.

The spherical to cylindrical shape with a diameter of approximately 2 mm suggests that the granules were produced by hot die pelletizing after compounding. Due to this complex and irregular shape, a precise and direct measurement of the resistance is impossible. Therefore, the granules are reshaped. The granules are compressed between two preheated plates to a thickness of 230 µm. The forming process takes place at a temperature of 220 °C and the granule is placed between the plates immediately before the forming process. From the insertion of the granules to the removal of the formed sheet, the entire forming process takes less than 10 s. The edges of the sheet are then trimmed with scissors, leaving a 6 mm wide strip, see Figure 5a,b.

On the specimen’s surface, two 1 mm wide strips of silver paste are applied, maintaining a 6 mm gap between them. The resistance of the sample is determined through a 2-wire measurement method, necessitated by the sample’s small dimensions. Through a 4-wire measurement procedure, compensation for contact resistance between the silver paste and the spring-loaded contacts, as well as lead resistance, is achieved. It should be noted that the contact resistance between the silver paste and the polymer remains uncompensated. Individual spring-loaded contacts are employed to establish contact for the sense and force lines. The measurement of height, utilized in resistivity calculations, is executed using a micrometer screw to account for any deviations from the nominal specimen height. Validation of the specimen proves unfeasible due to its small size.

#### 4.2.2. Measurement of the Filament Resistivity

**Figure 6:**
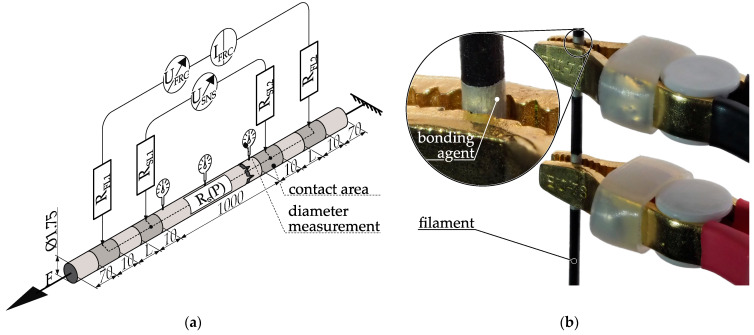
Measurement setup for determining the resistivity of filament by using 4-wire measurement: (**a**) schematic illustration and wiring of the experimental setup for filament specimen. R_S_(P) = resistance of the specimen depending on the parameters used to manufacture the filament, R_FL_= resistance force lead, R_SL_ = resistance sense lead; I_FRC_ = forced current, U_FRC_ = voltage of forced current, U_SNS_ = measured voltage drop across sample, L = distance between contact areas coated with electrical bonding agent, F = applied force. Specimens with L = 10 mm are used to characterize the process influences of filament production on resistivity; (**b**) one side of a filament specimen with a contact distance L = 10 mm.

For the characterization of the feedstock for material extrusion, the sample geometry and the adaptations of the procedure according to ISO 3915 are described [43]. Specimens for testing are directly created from filament sections. In Figure 6a, the specimen geometry is presented alongside the measurement setup utilized for determining its resistivity. Figure 6b displays one end of a real specimen featuring applied contacts and connectors.

The use of filament as the specimen eliminates the influence of subsequent process steps, such as thermal reshaping, on material properties. To minimize contact resistance, 10 mm wide contacts composed of silver paste are applied to the filament, with a 1000 mm spacing between the sense contacts. Resistance measurement is made with a 4-wire method using the Keithley 2460 sourcemeter. The outer contact strips supply the measuring current via the force leads, while voltage drop is measured via the inner ones via the sense leads. This 4-wire approach compensates for lead resistances R_FL1,2_ and R_SL1,2_. Additionally, the use of individual contact strips for force and sense compensates for contact resistance between connectors and the bonding agent, as well as from the bonding agent to the specimen surface. This measurement setup enables the accurate determination of the process dependent resistance R_S_(P). The diameter of the filament is measured in close vicinity to the upper and lower sense pads, as well as at the center of the filament piece, using a micrometer screw. Any deviations from the nominal diameter are recorded and either considered in the calculation or lead to the sample being designated as scrap. The average cross-section area of the filament is calculated from these individual measurements, and the resistivity of the filament is calculated using Equation (2). Compared to ISO 3915, significant modifications arise from the sample geometry (see Figure 6) [43]:

- Due to filament storage on spools, the filament retains the spool’s shape even after unwinding. To address this, the prepared filament piece is vertically hung with a 10 N weight pretension, aligning it without curvature and preventing contact between windings.
- The round geometry of the filament results in circumferential electrodes and necessitates an adjustment in their spacing. Furthermore, planar linear electrodes cannot be employed.

These adjustments necessitate the validation of the modified specimen. To evaluate the effect of pretension stress on the resistance, the pretension forces are adjusted, with a range up to 10 N. Within the examined range, the polymer composite ALFAOHM displays no notable piezo resistive characteristics.

**Figure 7:**
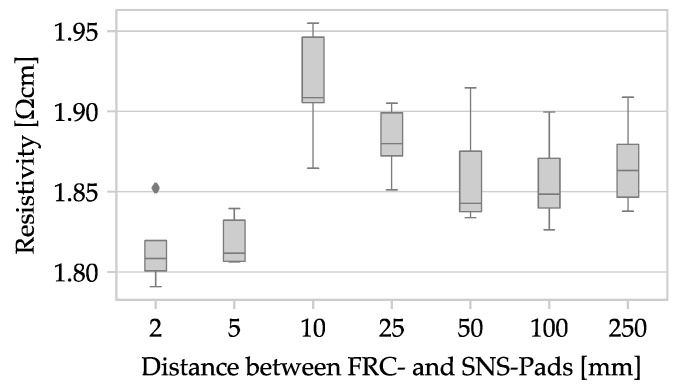
Box plot with the resistivity as a function of the distance L between the contact surfaces of filament specimens.

The electrical behavior during resistance measurement is impacted by altering the sample geometry from a plate to a rod, as well as by making changes to the shape and spacing of applied contacts. The distance between the measuring contacts is kept constant at 1000 mm to counteract geometric deviations. Additionally, diameter measurements at three points act as another measure to address geometric variations. To ensure a consistent current density across the sample’s cross-section at the measuring contact, a certain distance is necessary between the force and sense contacts, which is dependent on the polymer compound’s conductivity. In accordance to ISO 3915, a minimum distance of 30 mm is required for resistivity measurements up to 10^6^ Ω cm [43]. However, the ALFAOHM filament has a resistivity under 10 Ω cm, enabling a decrease in this distance between force and sense pads. Nonetheless, the specimen is validated for different distance variations, such as 2, 5, 10, 25, 50, 100, and 250 mm, with five samples taken for each distance variation. Figure 7 shows the evaluation results.

A significant effect of the distance parameter (L) on measured resistivity would be evident in a noticeable trend among the measurement results. Nevertheless, the analysis of the sampled materials demonstrates mean values that vary from 1.819 ± 0.015 Ω cm (L = 5) to 1.915 ± 0.036 Ω cm (L = 10). Notably, no clear pattern can be observed in this dataset. This observation indicates that the current density is likely to be evenly distributed either at the contact surfaces or immediately after, mainly due to the high conductivity of ALFAOHM. To simplify the application, a contact distance of L = 10 will be used for all future samples.

#### 4.2.3. Measurement of the Resistivity of Material Extrusion Specimen

Specimens are fabricated from the filament using MEX (see Figure 3). Planar monolayers are used for electrical characterization of the samples. In the case of multilayer samples, it is difficult to interpret the measurement result due to the resistance network formed by the contact resistances between the layers and the strand resistance within the strands. Single-layer samples are therefore used.

The orientation of the infill pattern has a significant impact on the resistivity [15] and current flow [1] within a layer. Infill patterns oriented at 0° or 90° can be used to achieve a uniform current distribution [1]. The aim of this study is to determine the factors that have an influence on the resistivity within polymer composites during the MEX process. In samples with a 90° infill pattern orientation, the current flows perpendicularly to the strands, creating a contact resistance that must be overcome at each strand interface. In contrast, with a 0° infill pattern orientation, the current flows parallel to the strands and the resistance is mainly influenced by the conductive properties of the strands. Since contact resistance can be influenced by other factors, 0° orientation reduces this error source within the sample. Additionally, the samples are produced without shells, as their removal decreases the complexity of the resulting resistor network.

**Figure 8:**
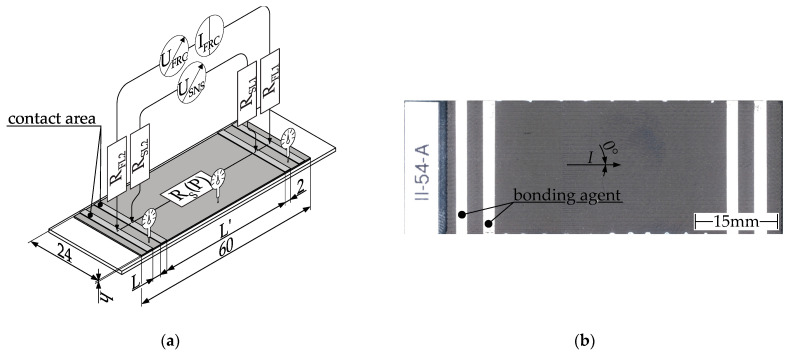
Measurement setup for determining the resistivity of MEX specimen by using 4-wire measurement: (**a**) schematic illustration and wiring of the experimental setup for MEX specimen. R_S_(P) = resistance of the sample depending on the MEX parameters, R_FL_= resistance force lead, R_SL_ = resistance sense lead; I_FRC_ = forced current, U_FRC_ = voltage of forced current, U_SNS_ = measured voltage drop across sample, L = distance between contact areas coated with electrical bonding agent, L’ = distance between sense contacts, h = height of specimen. Specimens with L’ = 10mm and L = 2 mm are used to characterize the process influences of MEX parameters on resistivity; (**b**) MEX specimen with applied bonding agent.

Removing firmly attached components from the build platform leads to high mechanical stress in the specimens that could affect the resistance. Specimens are therefore fabricated directly on glass slides, and all subsequent (characterization) procedures are performed as a combined glass/plastic specimen. Figure 8a illustrates the specimen with the measuring setup to determine the resistance. The height (h) of the specimen is an input variable in the DoE B. Figure 8b displays a physical MEX specimen with contacts of electrical bonding agent applied. 

Four 2 mm wide strips of silver paste are applied to the sample across its entire width, perpendicular to both the current flow and infill pattern orientation. This measurement setup of the MEX specimens is similar to the characterization of the resistivity of the filament using a 4-wire measurement technique. The contact between the samples and the sourcemeter is established using eight spring-loaded contacts (grid size: 2.54 mm) on each contact zone. These contacts consider surface irregularities during the MEX production process. The spring-loaded contact pins provide contact at each individual contact point. The micrometer screw measures the height of each sample with respect to the thickness of the slide before applying silver paste at both ends and the middle of the specimen. Specimens deviating by more than 5% from the nominal height were scraped. Notably, adjustments were made to the specimen geometry when compared to ISO 3915 [43].

- Spring-loaded contacts are used instead of planar line-contacted electrodes to compensate for surface irregularities.
- The specimen consists of only one layer (100/150/200 µm) and is therefore much thinner than the 3–4 mm range recommended by ISO 3915. This removes the effect of interlayer contact resistance during electrical characterization [43].
- The sense pad spacing is increased to average any manufacturing inaccuracies in the MEX process. Due to the restrictions of the slide dimensions, the force, and sense contacts are set closer together, with a smaller distance selected.

**Figure 9:**
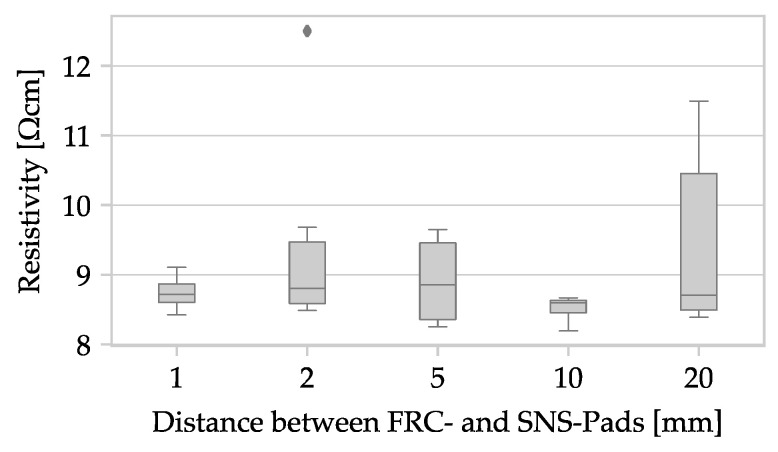
Box plot with the resistivity as a function of the distance L between the contact surfaces of MEX specimens.

The reduction in specimen thickness and the positioning of the contacts may cause the current density at the measuring point in the specimen to diverge from ISO 3915 requirements [43]. It is therefore necessary to validate the specimen geometry and contacts. During validation, the distance L’ between the two sense contacts remains a constant at 10 mm, while the distance L between the force and sense contacts is varied. The distances examined are 1, 2, 5, 10, and 20 mm, and five samples are measured for each distance variation investigated. See Figure 9 for the evaluation results.

Similar to the filament samples, a significant influence of the distance L on the measured resistivity would result in a noticeable trend in the measurement results. The examined specimens show mean values in the range of 8.50 ± 0.199 Ω cm (L = 10) to 8.766 ± 0.599 Ω cm (L = 5). The graph in Figure 9 shows minimal variation and is therefore unaffected by the input factor L. The distance between the contact surfaces has no effect on the measured resistivity of ALFAOHM. The distance L between force and sense contacts can be freely chosen and is selected as L = 3 mm for technical reasons of silver paste application. The distance between the sense contacts is set to L’ = 42 for all subsequent specimens in order to maximize the use of the slide length and thus increase the averaging.

### 4.3. Thermal Imaging

The 4-wire measurement provides an overall representation of the resistivity between the contact zones. However, this method cannot evaluate resistivity uniformity in the specimen.

Thermal imaging can identify areas of high-energy conversion. Equation (3) illustrates that the dissipated power P is proportional to the square of the voltage drop U and inversely proportional to the resistance R.

(3) $$P=\frac{U^{2}}{R}$$

**Figure 10:**
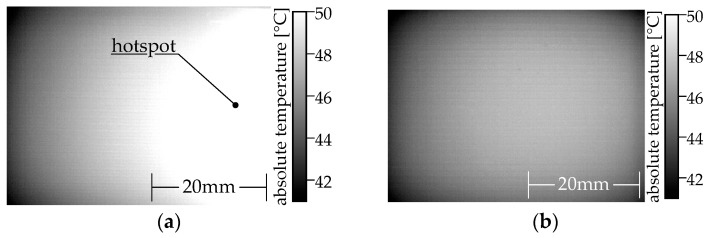
Comparison of two MEX monolayer specimens made from two different commercial filaments using the same MEX parameters: (**a**) inhomogeneous heat distribution (Protopasta conductive PLA); (**b**) homogeneous heat distribution (BlackMagic conductive).

The local temperature will increase as the local power density increases. A Microepsilon TIM640 thermal imaging camera is used to capture the heat distribution. The camera has a standard 33° optics. In order to ensure the comparability of the thermal images, the measurements are made with a constant power of 1 W. A snapshot is taken once the warmest pixel reaches 45 °C. This ensures that the heat deflection temperature of ALFAOHM is not exceeded. Two specimens made of two different polymer composites are shown in Figure 10 as references for the interpretation.

The left-hand (see Figure 10a) PLA/carbon black composite (ProtoPasta conductive PLA) specimen has a vertical hotspot, even though the process parameters are identical for both specimens. In contrast, the heat distribution of the right specimen (see Figure 10b) made of a PLA/graphene/carbon fiber/carbon black composite (BlackMagic conductive) is homogeneous. The temperature remains marginally lower in the peripheral areas.

**Figure 11:**
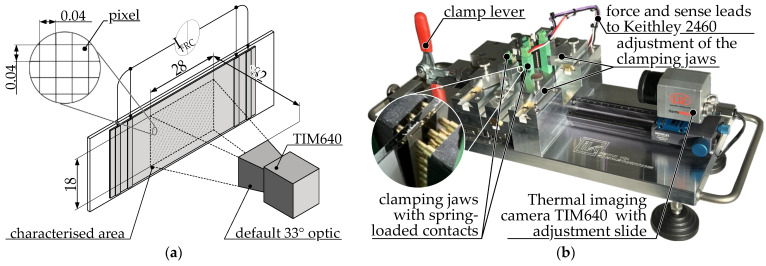
Measurement setup for the thermography of MEX specimen: (**a**) schematic illustration, wiring and positioning of the thermal imaging camera. A constant power is applied to the sample between the contact pads to generate heat. I_FRC_ = forced current; (**b**) test rig for repeatedly connecting the specimens to the sourcemeter and positioning them in front of the thermal imaging camera. The test rig is used to measure the resistance of MEX specimens and is also used for thermography.

Figure 11a displays the schematic arrangement for thermography. For a distance of 82 mm from the specimen, the pixel edge length on the specimen is 40 µm. The camera is an addition to the test rig shown in Figure 11b, for characterizing resistivity by 4-wire measurement. A clamping lever mechanism is used to position the sample in the focal plane of the camera. The sample is reproducibly positioned between clamping jaws made of spring-loaded electrical contacts and electrically connected to the sourcemeter. An adjustment slider can be used to fine tune the focal plane of the thermal imaging camera.

### 4.4. Scanning Electron Microscopy

Images of chosen specimens were captured using a scanning electron microscope (SEM). The specimens were cryogenically cooled with liquid nitrogen and then brittle fractured. All specimens were coated with a 4nm thick layer of platinum by sputtering to avoid charging on the fractured surface. A Helios G4 CX (Field Electron and Ion Company (FEI), Hillsboro, OR, USA) was used for taking the images. The images were captured using the secondary electron detector at 3 to 5 keV.

## 5. Results

The aim of this study is to determine the influence of selected process parameters of the filament production process and the MEX process on the resistivity of the material ALFAOHM. Different sets of process parameters (see Table 4) are used to manufacture filaments from the sourced granules. MEX specimens are fabricated and characterized using the most and least conductive in-house and commercial filaments. The results are compared in the following.

### 5.1. Filament Manufacturing

The filament production forms the starting point of this study. In this section, the results of the electrical characterization of the feedstock and the SEM images are presented.

#### 5.1.1. Resistivity and Additive Distribution of Granules

The electrical resistance of five granules was measured using the measurement procedure (see Section 4.2.1). The calculated resistivity is 2.46 ± 0.40 Ω cm.

**Figure 12:**
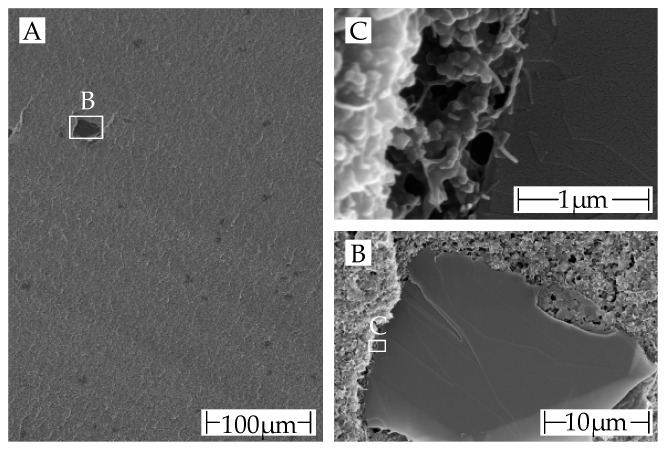
SEM images of the cryo-fracture edges of the granules: (**A**) macroscopic view of a large area of the granule. Darker areas are graphite; (**B**) magnification of a graphite particle; (**C**) edge of graphite particle with visible carbon black aggregates (spherical structures) and carbon nanotubes (tubular structures).

An imaging technique is used to evaluate the distribution of the additives (see Section 4.4). Figure 12 shows SEM images with different magnifications of the cryo-fracture surface of a granule.

The granules were manufactured by FILOALFA^®^ using hot die cutting, and the extrusion direction was not respected during cryo-fracturing. On the fracture surface are only a few darker areas (B) visible. They can be assumed to be graphite, as the scale of these darker areas matches that of the graphite particles in SEM images shown later. Although the datasheet only mentions carbon nanotubes as a conductive additive [6]. It can be concluded that the fracture surface is not perpendicular to the extrusion direction, as the fracture surface in SEM granules is clearly different from the fracture surface observed in the images of filament pieces shown later. CNTs and CB are also visible on the fracture surface of the highest magnification plane (C).

#### 5.1.2. Geometrical Accuracy and Filler Distribution of the Filament

**Figure 13:**
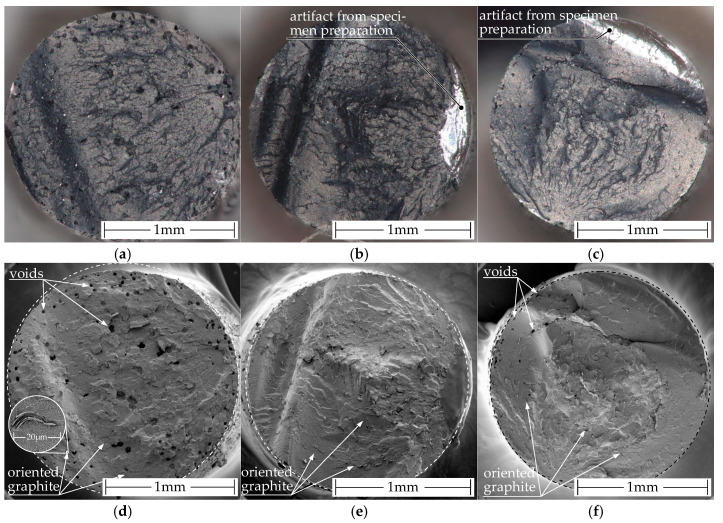
Macroscopic comparison of the cryo-fracture edges of the processed filaments. The images above were taken with an optical microscope (**a**–**c**): (**a**) commercial filament; (**b**) in-house manufactured filament with the highest resistivity; (**c**) In-house manufactured filament with the lowest resistivity. The images below (**d**–**f**) were taken with a SEM: (**d**) commercial filament; (**e**) In-house manufactured filament with the highest resistivity; (**f**) in-house manufactured filament with the lowest resistivity.

Figure 13 shows the fracture patterns of the filaments that were used for further specimen manufacturing. All specimens were cryo-fractured from their respective filaments.

Figure 13a,d show the fracture pattern of the commercial filament. In its border region, it contains many small voids, while in the core of the filament, there are fewer but larger voids. Figure 13b,c,e,f show the fracture patterns of the in-house-produced filaments. The filament shown in Figure 13b,e is the filament with the highest resistivity, and in Figure 13c,f the filament with the lowest resistivity. The filament with lower resistivity is the one that has a few pores. Evaporation of residual moisture in the granules during the extrusion process may have caused these voids. The granules used for in-house production were previously dried at 60 °C for 48 h. FILOALFA^®^ may have processed the granules at a higher residual moisture level. Differences in void formation between the two in-house productions may be due to different extrusion temperatures. The filament with a lower resistivity was extruded at a temperature of 200 °C, whereas the filament with the highest resistivity was extruded at a temperature of 180 °C and at the same screw speed. 

These two effects work together to have a self-reinforcing effect. Firstly, it increases the water vapor pressure in the polymer melt. Secondly, it decreases the viscosity of the polymer melt. 

The pressure drops from the pressure inside the die zone of the extruder to the ambient pressure as the polymer melt leaves the extruder. To achieve a state of force equilibrium between the pressurized voids and the new ambient pressure, the gas expands and enlarges the voids until they solidify. Polymer melts with low viscosity are less resistant to this deformation.

Consequently, residual moisture in the pellets increases the number and size of voids as extrusion temperatures rise. 

**Figure 14:**
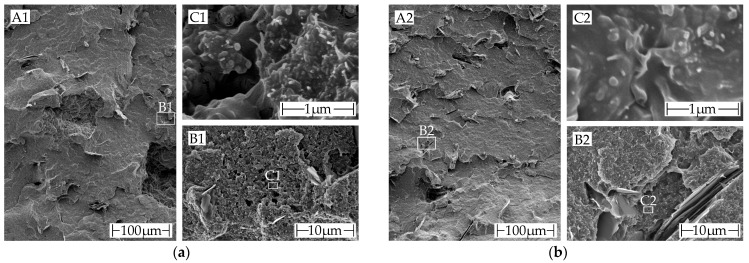
Microscopic comparison of the cryo-fracture edges of the commercially and in-house produced filaments: (**a**) commercial ALFAOHM filament: (**A1**) macroscopic image of the commercial filament fracture surface. Larger graphite particles can be seen protruding from the surface; (**B1**) magnification of an area with a graphite particle; (**C1**) magnification at which carbon nanotubes and carbon aggregates are visible; (**b**) in-house filament: (**A2**) macroscopic image a fracture surface of of in-house produced filament. Larger graphite particles can be seen protruding from the surface; (**B2**) magnification of an area with a graphite particle; (**C2**) Magnification at which carbon nanotubes and carbon aggregates are visible.

The orientation of graphite particles in the polymer melt is along the extrusion direction for all filament pieces visible (see Figure 13d–f). As a result, the graphite particles are oriented perpendicular to the fracture surface and become visible as brighter lines within the polymer matrix. The orientation is in Figure 14a,b better visible due to the higher magnification.

The fracture surface of both the commercial and in house filaments have a similar appearance at all magnification levels. At the lowest level of magnification, graphite can be observed perpendicular to the fracture surface (see Figure 14(aA1,bA2)). At the highest level of magnification, carbon black and CNTs are visible (see Figure 14(aC1,bC2)). The additives are evenly distributed and thoroughly integrated into the polymer matrix.

#### 5.1.3. Resistivity of Filaments Depending on Manufacturing Parameters

**Figure 15:**
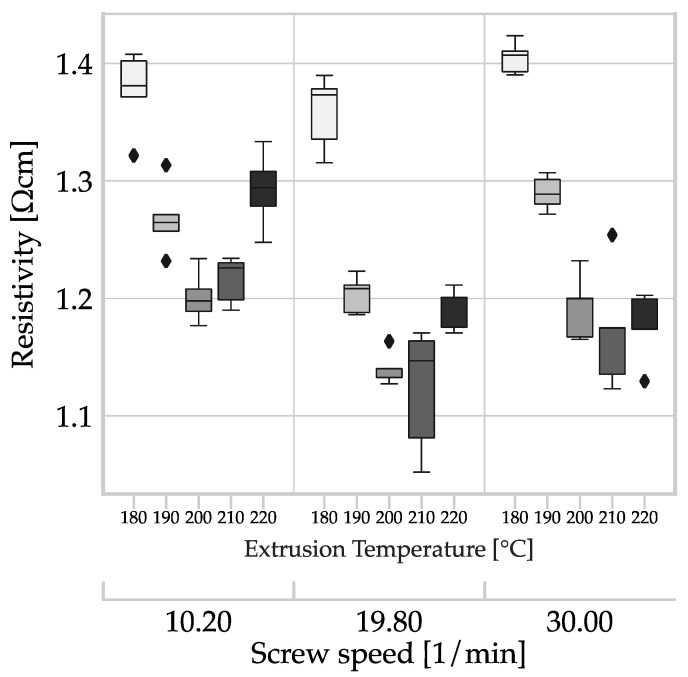
Box plot of the resistivity of filament as a function of the process parameters screw speed and temperature profile. The same color is assigned to data sets produced at the same extrusion temperature.

According to DoE A five filament sections from each batch of filaments are used as specimens for the measurement of the resistivity. The results are shown in Figure 15. The primary grouping is based on the influence of screw speed. Each group is subdivided by the extrusion temperature in the die zone.

The resistivity curve of each group is parabolic and is dependent on the extrusion temperature. In the range of parameters studied, an increase in resistivity is observed at both low and high temperatures, with the minimum in between. The screw speed has a smaller impact. The highest repeatable conductivity is achieved at an average screw speed of 19.8 L/min and an extrusion temperature of 200 °C.

The plot can be explained by superimposing effects due to the temperature dependent viscosity of the polymer melt, dispersion, degradation, and orientation of conductive additives consisting of carbon black and CNTs. Carbon black is present aggregates of carbon particles that provide good conductivity. Mechanical stress, particularly shear, breaks the bond between individual carbon particles in the aggregate. Initially, the conductivity increases due to dispersion, but long-lasting processing or strong shearing during the processing reduces the conductivity of the carbon black phase. The carbon particles are then only present as a loose agglomerate of primary particles [45]. Van Bellingen et al. established this parabolic conductivity curve over screw speed for a polymer composite with carbon black [46]. In a stress-free state, CNTs create tangles, resulting in a small air-line distance between the two ends of a CNT. External influences, such as shear in the polymer composite melt, cause the CNTs to align in response to their high aspect ratio. This results in an increase in the air-line distance between the ends of the CNTs [47,48,49]. The orientation along the extrusion direction leads to CNTs forming a highly conductive network across greater distances. The impact of shearing on the orientation of carbon black in the polymer melt is minimal, mainly serving to help establish contact between CNTs. Thereby enhancing the overall conductivity of the polymer composite [50,51].

The screw speed affects the shear within the polymer melt. Low screw speeds result in low shear, leading to minimal conversion of carbon black aggregates into agglomerates and, therefore, maintaining conductivity [45]. Simultaneously, the CNTs align themselves to a lesser extent, resulting in limited improvement in synergy with carbon black [47]. However, high screw speeds lead to high shear in the polymer melt, resulting in a high conversion rate of the carbon black aggregates into less conductive agglomerates [45,52]. Simultaneously, increased alignment of CNTs leads to improved conductivity. The alignment of conductive additives with high aspect ratios can be observed explicitly in Figure 14, notably with the graphite particles. Despite being bigger, they do not augment the conductivity.

Furthermore, the temperature affects the shear forces by altering the viscosity of the polymer melt. As the temperature increases, the viscosity of the polymer melt decreases, resulting in the retention of more carbon black aggregates during processing. However, this can lead to higher mobility of the carbon black particles, causing the existing conductive networks to break up, causing a decrease in conductivity. 

The combination of all factors leads to a minimum of resistivity at an extrusion temperature between 200 and 210 °C and a screw speed of 19.8 rpm. 

Table 5 presents measurement results of resistivity for granulate, commercial filament, and the most and least conductive in-house production. We conducted five measurements from one roll and one measurement from each of 22 rolls for the commercial filament. The granule samples have the highest resistivity and standard deviation.

The process of forming granules into sheet specimens is comparable to the process of compression molding. Thus, the resistivity of the sheet specimen is after the reshaping isotropic [53]. During the subsequent filament extrusion process, the conductivity may increase or decrease depending on the extent to which the carbon black and CNTs are uniformly dispersed in the sourced granules [45,54]. The slightly higher conductivity of the filament can therefore be a consequence of the orientation of the CNTs in the filament due to the extrusion and/or further dispersion of the carbon black in the polymer melt.

The resistivity of the roll used to fabricate the MEX samples was in the middle range of the 22 characterized rolls. The standard deviation across these 22 rolls is approximately 3% of the average resistivity. The two in-house-produced filaments have significantly lower resistivity compared to the sourced filament. This may be due to the higher material throughput and increased shear forces during the industrial scale production of FILOALFA^®^. A possible explanation for the differences in resistivity can be found in the interaction of the following mechanisms. In addition to carbon black, ALFAOHM also contains CNTs. In extrusion processes, high aspect ratio conductive additives such as CNTs are aligned along the shear direction [47,48,49]. This improves the conductivity in the extrusion direction [50]. In this study, resistivity was also measured along the extrusion direction. The carbon black phase is oriented to a lesser extent by shear and provides electrically conductive cross-linking of the CNTs [5]. Too much shear converts the carbon black aggregates into less conductive agglomerates [46], reducing the resulting conductivity of the filament.

This study aims to investigate how the process parameters during filament production affect the filament conductivity. In addition, the effect of the manufacturing parameters of the filament on the conductivity of MEX structures will be investigated. Therefore, the two in-house-produced filaments with the least and most conductivity are used to fabricate the MEX specimens. Table 6 provides the two process parameter sets for filament production and their conductivity.

### 5.2. Material Extrusion Results

MEX specimens are fabricated from the produced filaments. In the following, the results of these MEX specimens are evaluated in more detail.

#### 5.2.1. Geometrical Accuracy of the Material Extrusion Specimens

The calculation of resistivity requires a precisely known cross-sectional area. The MEX parameter of the flow rate influences the cross-section by determining the amount of extruded plastic. Therefore, the flow rate must be adjusted individually for each set of MEX process parameters. These include, in particular, filament diameter, extrusion temperature, deposition rate, and layer height. The flow rate must be adjusted due to the difference between the actual filament diameter and the filament diameter used by the slicer during trajectory planning. The extrusion temperature as well as the deposition speed will have an effect on the flow behavior of the polymer melt and will therefore have to be adjusted accordingly. 

**Figure 16:**
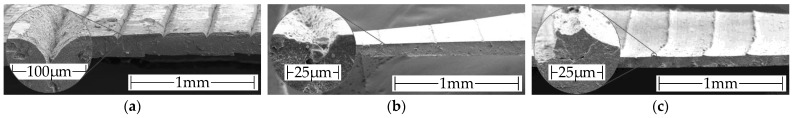
SEM images of cryo-fractured specimens with different surface qualities: (**a**) specimen with an under-extrusion; (**b**) specimen with ideal flow rate; (**c**) specimen with over-extrusion.

The flow rate was adjusted according to the surface roughness based on appearance and by measuring with a micrometer screw. The flow rate was adjusted until a uniform surface of the MEX samples was obtained with minimal under-extrusion. Figure 16 shows cryo-fracture images of three specimens at different flow rates.

Figure 16a shows a specimen with under-extrusion. Distinct notches between the strands characterize the surface of the specimen. This means that the cross-sectional area for current flow is reduced compared to the rectangular area assumed for the resistivity calculation (2). Extruding too much material is known as over-extruding. Excess extrusion results in material peaks on the otherwise flat surface of the sample, as shown in Figure 16c. This increases the conductor’s cross-section. An example of the fracture profile of a specimen with an acceptable surface finish is shown in Figure 16b. For each parameter set, the flow rate has been set to produce the results shown in Figure 16b. Another source of error is the positioning variation of the nozzle relative to the build platform. This causes the actual geometry of the specimen to deviate from the nominal geometry. Possible causes could include thermal deformation and wear observed in the linear guides, among other factors. Despite extensive adjustments to the system, errors cannot be completely ruled out. For this reason, the thickness of each sample was measured at three points immediately after removal from the building platform using a micrometer screw. A maximum deviation of 5% from the nominal thickness of the sample is used as a rejection criterion.

**Figure 17:**
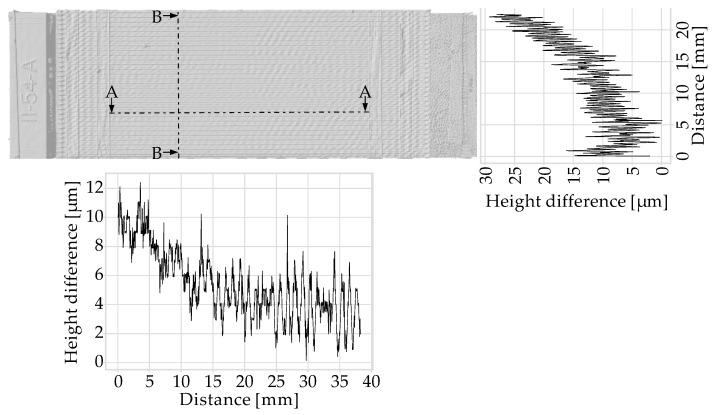
3D Scan of MEX specimen with two height profiles derived from it. The A-A profile runs parallel to the deposition direction, whilst the B-B profile runs perpendicular to the deposition direction.

Due to the time involved, only a few samples were examined using the Keyence VR-5100 optical surface profilometer. Figure 17 shows an example of a 3D Scan with two derived cross-section profiles.

Profile B-B run perpendicular to the strands and exhibit a drift so that the specimen is slightly thicker on one side than on the opposite side. Due to notches between the strands caused by slight under-extrusion, higher frequency height differences are superimposed on the profile.

Profile A-A runs centrally on one strand. The micrometer screw has a low-pass filter effect due to the 6.5 mm diameter measuring surfaces and the applied force during the measurement. As a result, the readings at the three measurement points may deviate from the average sample thickness.

#### 5.2.2. Influence of Material Extrusion Process Parameters on the Resistivity

**Figure 18:**
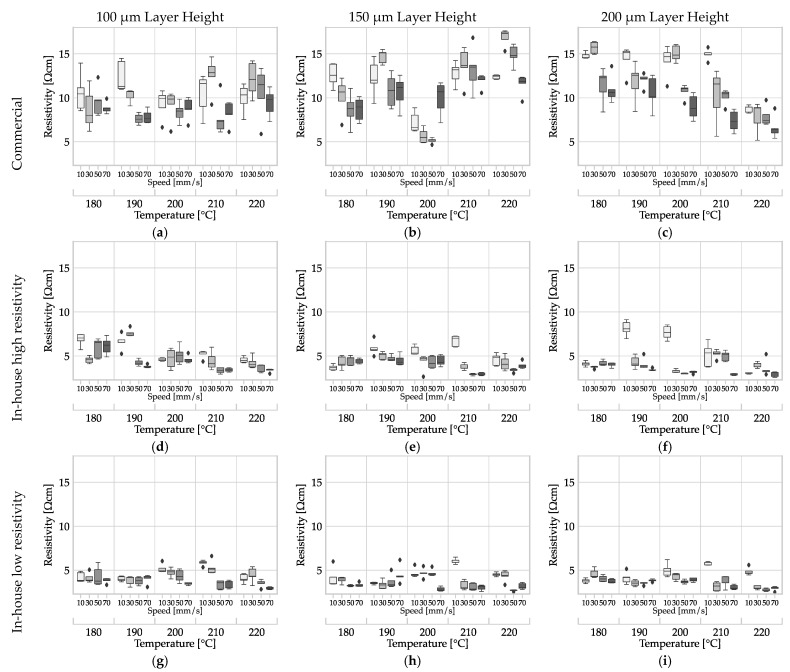
Influence of the input factors of (deposition) speed, (extrusion) temperature, layer height and filament on the resistivity of monolayers of ALFAOHM produced by MEX: (**a**–**c**) resistivity of MEX structures made of commercially sourced filament with layer heights of 100 µm (**a**), 150 µm (**b**) and 200 µm (**c**); (**d**–**f**) resistivity of MEX structures made of in-house-produced filament with the highest resistivity with layer heights of 100 µm (**d**), 150 µm (**e**) and 200 µm (**f**); (**g**–**i**) resistivity of MEX structures made from in-house-produced filament with the lowest resistivity at layer heights of 100 µm (**g**), 150 µm (**h**) and 200 µm (**i**).

The resistivity data are plotted in Figure 18, showing its dependence on temperature, deposition rate, layer height, and filament. In each subplot, the two input factors (extrusion) temperature and (deposition) speed are combined. The sub-plots are arranged according to the input factors, with rows corresponding to the filament used and in columns according to the layer height. All plots are scaled the same to enable direct visual comparison of data. 

A comparison of the data within a row for any of the filaments in Table 5 indicates that layer height has no impact on resistivity. The input factors, deposition speed and extrusion temperature, also have no substantial impact on resistivity. Watschke et al. reported comparable results for filaments filled with CNT with an infill orientation of 0° [15]. The resistivity determined for commercial filament MEX structures surpasses the manufacturer’s specification of 15 Ω cm within layers [6].

The average resistivity of all specimens from the commercial filament is 10.67 Ω cm; 4.49 Ω cm for the least conductive in-house production, and 3.94 Ω cm for the most conductive. The average increase in resistivity across all samples from the commercial filament (1.697 Ω cm) through MEX processing to the MEX structure (10.67 Ω cm) is over 620%, whereas the average change in average resistivity from the least in-house-produced filament (1.401 Ω cm) to the MEX structures (4.49 Ω cm) is approximately 320%. For the most conductive in-house-produced filament (1.141 Ω cm), the average change compared to the MEX structures (3.94 Ω cm) is about 345%. However, it is evident that filament, or rather, the process parameters of filament production, have a significant influence on the resulting conductivity of MEX structures. The same observation has already been made by Dul et al. [55]. Furthermore, the standard deviation is lower for in-house production. 

The type of filament, whether commercial or in-house produced, is the main factor influencing the significant conductivity variation in the MEX samples. Due to the industrial scale of commercial filament production, the volume flow through the extruder was presumably higher. This increases the shear rate, shear stress, and pressure, leading to increased agglomeration of the carbon black aggregates. Abbasi et al. report this dependency [45]. The resulting loss of conductivity of the carbon black phase is compensated by the alignment of the CNTs [47]. Due to this mechanism, it is plausible that the commercial filament produced under high stress has a similar conductivity to the filament produced under lower stress on a laboratory scale. 

The shear rate in MEX extrusion is lower [46] and the time under shear is significantly shorter than in filament extrusion. As a result, the CNT alignment mechanism cannot compensate for the conductivity loss as it does in the filament production process [45]. The overall conductivity is reduced by the reduced orientation of the CNTs along the strand direction. For this reason, the reduction in the conductivity of the carbon blacks phase due to the conversion of aggregates to agglomerates as well as the dispersion has a greater effect.

#### 5.2.3. Thermal Images of Material Extrusion Specimens

**Figure 19:**
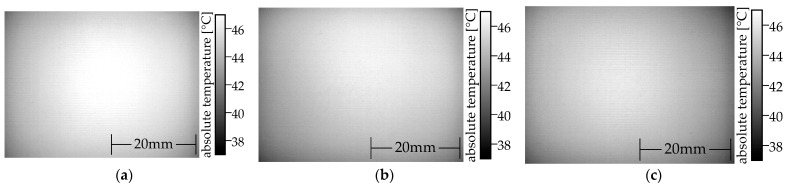
Thermographic images of MEX monolayer specimen from the three filaments analyzed. The MEX process parameters are identical for all samples. The current flows from left to right parallel to the strands: (**a**) specimen manufactured from commercial filament; (**b**) specimen manufactured from the least conductive in-house filament; (**c**) specimen manufactured from the most conductive in-house filament.

Thermography can be used to determine the uniformity of the local conductivity in MEX specimens. Any variation in local conductivity from the mean conductivity will result in a variation in current flow in the specimen and will change the local power dissipation. Figure 19 shows thermal images of MEX specimens. The same MEX process parameter set was used to manufacture all three specimens. The circular heat distribution is an imaging artifact of the used optic.

All thermal images display a homogeneous heat distribution, like specimens made of BlackMagic conductive in the center with slightly lower temperatures at the periphery (see Figure 10b), although the conductivity is decreased due to the PTC behavior of the carbon black phase in the polymer [56]. The carbon nanotubes (CNTs) have a high aspect ratio and can counteract the loss of conductivity resulting from the positive temperature coefficient (PTC) behavior of the carbon black. This compensation ensures that no local thermal runaways occur, unlike in Protopasta conductive PLA (see Figure 10a). 

## 6. Summary and Conclusions

The objective of this study was to comprehensively characterize the process-specific influencing factors along the MEX process chain, from filament production to MEX processing, on the resistivity of electrically conductive structures. The key findings for the processing of ALFAOHM composites include the following:

1. The resistivity of the filament can be influenced by the process parameters of the polymer extruder (screw speed and temperature profile).
2. The investigated MEX process parameters, namely layer height, deposition speed and extrusion temperature, have no significant effect on the specific resistance of electrical conducting MEX structures.
3. The resistivity of the MEX structures made from the filament cannot be trivially deduced from the resistivity of the filament.

As a first step, specimens were developed for the electrical characterization of granules, filaments, and MEX monolayers. These specimens were successfully validated in accordance with ISO 3915 [43]. A total of 15 sets of process parameters for filament production were investigated by varying the screw speed and the extruder temperature profile using ALFAOHM granules supplied by FILOALFA^®^. The resistivity of the in-house-produced filaments depends significantly on the extrusion temperature and only to a limited extent on the screw speed in the investigated parameter range, ranging from 1.141 ± 0.013 Ω cm to 1.401 ± 0.064 Ω cm. The resistivity is parabolically dependent of the extrusion temperature. The minimum resistivity of 1.141 ± 0.013 Ω cm is reached for the process parameters at 210 °C and 19.8 rpm. The commercial filament sourced from FILOALFA^®^ has a resistivity of 1.697 ± 0.052 Ω cm. To investigate the process parameters, the MEX process parameters, including extrusion temperature, deposition speed, and layer height, were varied during the specimen fabrication. Using the least and most conductive in-house and commercial filaments, 60 different sets of process parameters were used to fabricate specimens using MEX. The MEX process parameters were found to have a non-significant influence on resistivity. Therefore, the MEX process parameters are freely selectable for ALFAOHM. The significant difference between the conductivities of the MEX structures from the commercial reference filament (filament: 1.697 ± 0.052 Ω cm, MEX structure: 5–15 Ω cm) and the in-house fabrications (filament: 1.141 to 1.401 Ω cm, MEX structure: < 5 Ω cm) shows that the filament fabrication process parameters have a significant influence. As the full parameters set of the filament production of the reference material ALFAOHM at FILOALFA^®^ are unknown, no conclusive statement can be made at this stage as to the reasons for the differences in conductivity. However, it can be assumed that due to the higher volume throughput of industrial scale production, higher shear rates occur in the extruder, which has a significant influence on electrically conductive additives and therefore on conductivity.

Integrating conductive structures into multi-material product design remains the key objective. This necessitates extensive knowledge in areas such as material selection, process parameters, trajectory planning, electrical contacting, and design. The following recommendations for the design and manufacturing of products by means of MEX can be derived from the results obtained in the present study:

1. The investigated MEX process parameters (layer height, deposition rate and extrusion temperature) can be chosen freely, since they have no significant reproducible influence on the resistivity.
2. The resistivity used for the design of electrical functional structures should be determined using a MEX test specimen so that all influences along the manufacturing chain are taken into account.
3. The aim should be to achieve the lowest possible resistivity and high reproducibility (low scatter) through all process parameters along the process chain. This results in greater design freedom to geometrically influence the absolute structural resistance.

Future research will deepen this knowledge and develop practical guidelines for successfully implementing such integrations.

The integration of electrically additively manufactured conductive structures usually requires an interface to a conventional electrical system. This contact interface has requirements in terms of contact resistance, scalability, detachability, mechanical strength, etc. A design support in the form of a selection guideline would be useful to assist product developers.

In addition to using conventional filaments as feedstock, the MEX process also enables the use of granules. This approach removes the intermediary step of filament production and consequently the extrusion process, which, as demonstrated in this study, greatly affects the electrical material properties of MEX structures. MEX using granules as feedstock also offers opportunities for scalability and cost reduction [2]. An in-depth comparison between these two approaches in terms of their influence on the electrical properties should therefore be investigated in more detail.

Post-processing can affect conductivity. One example is the annealing of MEX structures, which temporarily increases the mobility of conductive additives in the polymer phase [19,56]. This affects the resulting resistor network.

## Figures and Tables

**Table 1:** Literature review on the effects of process parameters on the electrically conductive properties of structures by process steps of filament product production and MEX processing.

	Study	Podsiadły et al. [18]	Gonçalves et al. [19]	Dul et al. [16]	Zhang et al. [20]	Dorigato et al. [21]	Yang et al. [22]	Kwok et al. [23]	Masarra et al. [24]	Spinelli et al. [17]	Paz et al. [14]	Barsši Palmic et al. [25]	Gao and Meisel [26]	Watschke et al. [15]
material	commercially available	(◆)									(◆)	◆	◆	◆
	matrix polymer	ABS	PEEK	ABS	PLA	ABS	PVA	PP	PLA, PCL	PLA	ABS	PCL	PLA	PLA, PCL
	additive (legend below)	CNT	GnP, CNT	CNT	GO	CNT	GNP	CB	GnP	GnP, CNT	GnP	CP	CB, GnP	CB, CNT, CP
feedstock	filament fabrication	◆	◆	◆	◆	◆	◆	◆	◆	◆	◆			
	extruder temp. profile	⊛									⊛			
	screw speed										⊛			
	screw profile		⊛											
	nozzle geometry										⊛			
studied MEX parameters	layer height										⊛	⊛	⊛	
	deposition speed												⊛	⊛
	extrusion temperature						⊛					⊛	⊛	⊛
	build platform temp.													
	infill pattern													
	infill pattern orientation			⊛			⊛		⊛		⊛			⊛
	infill percentage													
	strand width										⊛			
	nozzle diameter	⊛										⊛		
	flow rate											⊛		⊛
	cooling													
characteri-sation	electrical bonding		Ag	Ag			Ag	Ag		Ag	Ag	Ag		Ag
	resistivity granules													
	resistivity filament		◉	◉						◉	◎			◎
	resistivity MEX specimen	◉		◉	◎	◉	◎	◎	◉		◎	◎	◎	◎
	SEM			◆	◆		◆	◆	◆					

Additives: CB = carbon black; CNT = carbon nanotube (single and multiwall CNT combined); GnP = graphene nanoplatelets; GO = graphene oxide; CP = copper particles. Electrical bonding: Ag = silver paste/epoxy. ◆ = true statement, ⊛ = varied parameters. Resistance measurement: ◎ = 2-wire measurement; ◉ = 4-wire measurement.

**Table 2:** Overview of commercially available electrically conductive filaments.

Material	Filler ^2^	Resistivity ^2^ [Ω·cm]	Matrix Polymer	Operating Temperature [°C]	Price [€/kg]	Availability (2022 Q4)
Multi3d Electrifi [27]	copper particle	0.006	PCL	55 ^2^	2050	◆
BlackMagic Conductive [28]	graphene, carbon fiber	0.6	PLA	50 ^2^	2000	
Functionalize F -Electric™ PLA [29]	CNT	0.75	PLA	50 ^1^	300	
Blackmagic Conductive flexible Filament [30]	graphene	<1.25	TPU 92A	-	800	
Amolen conductive PLA [31]	n.a.	1.42	PLA	50 ^1^	100	◆
Koltron G1 [32]	graphene	2	PVDF	100 ^2^	600	◆
Conductive Filaflex [33]	carbon black	3.9	TPU 92A		150	◆
Ampere PLA [34]	CNT	4	PLA	50 ^1^	118	◆
ALFAOHM [6]	CNT	15(xy)/20(z)	PLA	50 ^1^	260	◆
Protopasta conductive PLA [35]	carbon black	15	PLA	50 ^1^	100	◆
3dkonductive electroconductive [36]	carbon black	24	PLA	50 ^2^	110	◆
FILI conductor TPU [37]	carbon black	27.44	TPU	-	150	
PI-ETPU 85-700+ [38]	carbon black	<800	TPU 95A	-	160	
Eel 3D Printing Filament [39]	n.a.	1500	TPU 90A	-	140	◆

^1^ Estimation of the operating temperature of the matrix polymer. ^2^ According to manufacturer specifications. ◆ = available in 2022 Q4.

**Table 3:** Overview with the process parameter range recommended by the manufacturer for the material ALFAOHM (granules and filament).

Parameter	Manufacturer Recommendation	
	Lower Limit	Upper Limit
extrusion temperature [°C]	190	210
build platform temperature [°C]	0	50
deposition speed [mm/s]	10	50

**Table 4:** Design of Experiment for filament production and MEX specimen fabrication.

Production Steps	Design of Experiment Input Factor	Lower Limit	Upper Limit	Incre-ment	Filament Commercial	Filament in House
filament production (DoE A)	temperature die zone [°C]	180	220	10		◆
	screw speed [rpm]	10	30	10		◆
MEX-TRB/P/PLA (DoE B)	extrusion temperature [°C]	180	220	10	◆	◆
	deposition speed [mm/s]	10	70	20	◆	◆
	layer height [mm]	0.1	0.2	0.05	◆	◆

◆ = Input factor analysed.

**Table 5:** Comparison of resistivity of granules and filaments produced by different methods.

Specimen	Average Resistivity [Ω cm]	Standard Deviation [Ω cm]
ALFAOHM granules (hot pressed)	2.462	0.403
ALFAOHM commercial filament (one spool)	1.708	0.012
ALFAOHM commercial filament (22 spools)	1.697	0.052
ALFAOHM highest-resistivity in-house filament	1.401	0.064
ALFAOHM lowest resistivity in-house filament	1.141	0.013

**Table 6:** Filament manufacturing process parameters of the two in-house-produced filaments further used.

Parameter	In-House Lowest Resistivity (1.1 Ω cm)	In-House Highest Resistivity (1.47 Ω cm)
Temp. die zone [°C]	200	180
Screw speed [rpm]	19.8	19.8

## Data Availability

The data presented in this study are available on request from the corresponding author.
